# A Systematic Review and Meta‐Analysis of Randomized Controlled Trials on the Benefits of Using Lactobacillus Supplements as an Adjunct Treatment for *Helicobacter pylori* Eradication

**DOI:** 10.1002/mbo3.70166

**Published:** 2025-12-01

**Authors:** Asma Azam, Muhammad Abdul Muqtadir Qureshi, Hafiz Shahbaz Zahoor, Syeda Malaika Raza, Muhammad Mohsin Khan, Umaimah Naeem, Syed Atif, Abdul Waheed

**Affiliations:** ^1^ Karachi Medical & Dental College Karachi Pakistan; ^2^ Quaid‐e‐Azam Medical College Bahawalpur Pakistan; ^3^ Jinnah Sindh Medical University Karachi Pakistan; ^4^ Mary Washington Family Medicine Residency Program Fredericksburg Virginia USA; ^5^ Department of Family Medicine Dignity Health Medical Group Arizona; ^6^ Creighton University School of Medicine Phoenix Arizona USA

**Keywords:** adjunct therapy, efficacy, helicobacter pylori, lactobacillus, placebo

## Abstract

Supplementing *H. pylori* treatment with probiotics like Lactobacillus has become an essential approach due to the possible adverse effects of antibiotic therapy and the need to increase overall eradication rates. Although several types of Lactobacillus strains as probiotics were efficient in treating *H. pylori*, their relative efficiency in treating *H. pylori* was uncertain. A survey of databases, including PubMed, Cochrane, Google Scholar, Scopus, and Clinicaltrials.gov, retrieved 52 Randomized Controlled Trials (RCTs), with 14 meeting the criteria for RCTs on Lactobacillus supplementation (LS) as an adjunct therapy compared to placebo in adult *H. pylori* patients. Analyses were conducted using RevMan5.3, Cochrane Risk of Bias Tool, Comprehensive Meta‐Analysis Software, and GRADEpro. Fourteen RCTs, including 2054 patients with more than ten different probiotics, were included in this analysis. The LS group showed significantly higher *H. pylori* eradication rates [RR = 1.04 (95% CI: 1.01, 1.07; *p* = 0.009; *I* ^2^ = 0%); (high certainty)], decreased AEs including vomiting [RR = 0.82 (95% CI: 0.48, 1.41; *p* = 0.48; *I* ^2^ = 19%); (high certainty)], diarrhea [RR = 0.45 (95% CI: 0.26, 0.80; *p* = 0.007; *I*² = 55%); (high certainty)], abdominal pain [RR = 0.73 (95% CI: 0.28, 1.93; *p* = 0.53; *I*² = 66%); (high certainty)], anorexia [RR = 0.79 (95% CI: 0.23, 2.64; *p* = 0.70; *I*² = 0%); (high certainty)], constipation [RR = 1.02 (95% CI: 0.42, 2.50; *p* = 0.96; *I*² = 0%); (high certainty)], rash [RR = 1.51 (95% CI: 0.57, 3.98; *p* = 0.41; *I*² = 0%); (high certainty)], taste disturbance [RR = 0.64 (95% CI: 0.44, 0.92; *p* = 0.02; *I*² = 51%); (moderate certainty)], and reduction of gastrointestinal symptoms including abdominal pain [SMD = −0.19 (95% CI: −0.46, 0.09; *p* = 0.18; I² = %); (moderate certainty)]. None of the included RCTs depicted a high risk of bias. Lactobacillus added to triple or quadruple therapy increased eradication rates, but improvements in adverse effects and gastrointestinal symptoms were not significant. Multiple different strains limited assessment of individual effectiveness, preventing firm conclusions about the specific impact of each Lactobacillus type.

## Graphical abstract

Lactobacillus supplementation, when used alongside triple or quadruple antibiotic therapy, significantly improved Helicobacter pylori eradication rates compared to placebo. Although reductions in adverse events and gastrointestinal symptoms were not statistically significant, strain variability limits conclusions regarding the specific efficacy of individual Lactobacillus types.

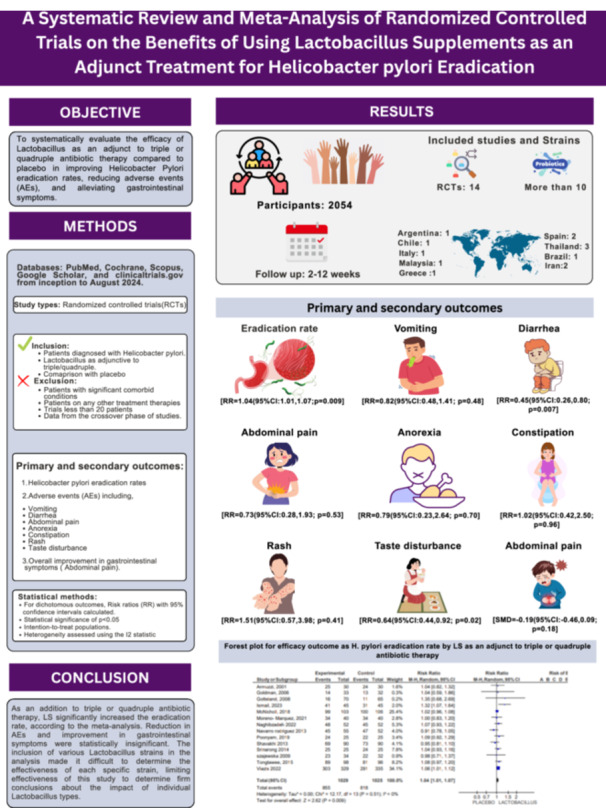

## 1 Introduction

MALT lymphoma, stomach adenocarcinoma, peptic ulcers, as well as chronic gastritis are all primarily linked to *Helicobacter pylori* (*H. pylori)* as the common causative factor. It has received plenty of attention from all across the world since it was discovered (Narayanan et al. 2018). Half of the world's population is affected by it (Itskoviz et al. 2017). This indicates that an estimated figure of 4.4 billion people are affected globally, and the infection rate is greater in developing nations. Nigeria makes the peak *H. pylori* burden at 87.7%, then comes Portugal with 86.4%, Estonia with 82.5%, Kazakhstan with 79.5%, as well as Pakistan with 81.0% (Hooi et al. 2017). Many people with *H. pylori* experience symptoms like dyspepsia, although some are asymptomatic. Patients, even those with minor symptoms, are increasingly being screened for *H. pylori* infections and receiving active treatment for them (Malfertheiner et al. 2002). *H. pylori* therapy is made more difficult by growing incidence of antibiotic resistance, and it is crucial to take into account how antibiotic therapy affects the gut microbiota (Zhou et al. 2022; Malfertheiner et al. 2022). The eradication rate has decreased in recent years, mainly caused by antibiotic resistance and adverse events (AEs) of different drugs which are mostly gastrointestinal in nature (Hu et al. 2016; Thung et al. 2016). The limitations of current therapies and the need to conserve the gut microbiome imply further research into the potential use of probiotics such as Lactobacillus in managing *H. pylori* infections (Suzuki et al. 2022).

A preparation containing live microorganisms that, when added to the diet, enhances the health of the host by promoting a balanced intestinal microflora (AFRC 1989). Several clinical trials report that with addition of probiotics in the eradication therapy a better therapeutic response and lower incidence of AEs in *H. pylori* eradication was achieved (LV et al. 2015; Gong et al. 2015). According to a different study that examined the impact of adding several probiotics in *H. pylori* eradication therapy, Lactobacillus and its numerous strains are good probiotic options. In this study, participants in China also showed a higher rate of eradication than those in other nations (Shi et al. 2019). In vitro studies suggest that Lactobacillus species can potentially suppress *H. pylori* growth in mice (Johnson‐Henry et al. 2004).

A study conducted in 2019 (Yu et al. 2019) was confined to studies utilizing standard triple therapy and evaluated only a limited set of adverse effects. Notably, among these, only taste disturbance yielded significant findings. Furthermore, the review was based on 11 studies comprising 972 patients, most from Europe. The authors acknowledged this geographical limitation and the restricted range of side effects considered. Additionally, gastrointestinal symptoms were not comprehensively assessed using a standardized instrument such as the GSRS, and the analysis was limited to five Lactobacillus species. Crucially, their search was restricted to studies published up until July 2019, leaving newer trials unexamined. In contrast, the present review offers a more expansive and up‐to‐date synthesis by including both triple and quadruple therapy regimens, analyzing a broader spectrum of Lactobacillus species, and incorporating studies published after 2019. Furthermore, this provides a more extensive evaluation of adverse effects and gastrointestinal outcomes. This comprehensive approach seeks to address the limitations of previous meta‐analyses, offering a more nuanced and inclusive understanding of the influence of Lactobacillus supplementation (LS) in the eradication of *H. pylori*.

## 2 Methods

### 2.1 Data Sources and Search Strategy

Starting and ending in August 2024, a detailed search was made across PubMed, Scopus, Cochrane, Google Scholar, as well as clinicaltrials.gov. The keywords and medical subject headings (MeSH) that were utilized are given as follows:

(Patients diagnosed with *Helicobacter pylori* OR “Helicobacter pylori”[Mesh]) AND (lactobacillus OR “Lactobacillus”[Mesh]) AND (placebo OR placebos OR “Placebos”[Mesh]) AND (outcomes OR efficacy OR effectiveness OR “Treatment Outcome”[Mesh])

PubMed was searched using both free text terms and MESH.

The PICO (Population, Intervention, Comparison, Outcome) criteria was employed to screen the studies to be included in our meta‐analysis included: patients (P) as diagnosed with *Helicobacter pylori*; intervention (I) as the use of various Lactobacillus adjunct therapy (LS); control (C) as patients on placebo; and outcomes (O) defined as the *Helicobacter pylori* eradication rates of, the rate AEs occuring which consists gastrointestinal symptoms, and the overall improvement in gastrointestinal symptoms. This study only included the English language trials that are RCTs in nature.

### 2.2 Selection Criteria

The inclusion standards in our analysis included: (i) RCTs that compared the impact of LS as adjunct therapy compared with placebo, (ii) studies that are based on participants diagnosed with *Helicobacter pylori*.

Exclusion criteria were as follows: (i) clinical trials that involved patients who had significant comorbid conditions or were on any other treatment therapies that could potentially affect *Helicobacter pylori* eradication or gastrointestinal outcomes, (ii) trials that had fewer than 20 patients per treatment group, (iii) data from the crossover phase of studies were excluded due to potential concerns regarding carryover effects.

### 2.3 Selection Process

Two independent authors scrutinized every paper's preliminary data and ensured that all studies in question fulfilled the inclusion requirements. Two independent authors also reviewed potential articles in full text. The salient features of the extracted data included the significant characteristics of the trials, the therapeutic designs, and the outcomes.

### 2.4 Outcome Measures

For analyses, *H. pylori* eradication rates, AEs including vomiting, diarrhea, abdominal pain, anorexia, constipation, rash, and taste disturbance, and the overall improvement in gastrointestinal symptoms constituted the outcomes. Data were extracted post‐intervention period, as reported by each study. For these outcomes, relative risks (RR) and standardized mean differences (SMD), along with their respective confidence intervals, were thoroughy extracted and added in the analysis.

### 2.5 Data Extraction

Extraction was independently executed by two of the individuals from our team via standard data extraction forms. As mentioned above, the pertinent graphs and tables were used to obtain data on the results. Patient variables and demographics (such as mean age, gender, and so on) from the many studies included in the analysis were presented in a data table. (Table 1).

**Table 1:** Summary of the characteristics of randomized controlled trials evaluating various *Lactobacillus* species supplementation as an adjunct therapy for *Helicobacter pylori* eradication.

First author	Year	Country	Patients exp/cont	Follow up line	Treatment eradication regimen	Type of probiotic/lactobacillus used	Eradication rate Exp/cont
Goldman	2006	Argentina	33/32	4 and 12th week	Amoxicillin (50 mg/kg/day), clarithromycin (15 mg/kg/day), omeprazole (1 mg/kg/day). For 1 week	Bifidobacterium animalis Lactobacillus casei.	42.42%/40.63%
Gotteland	2008	Chile	70/65	3 and 4 weeks	Cranberry juice (200 mL) 3 weeks with no administration on weekends.	Lactobacillus johnsonii La1 (La1).	22.86%/16.92%
Armuzzi	2001	Italy	30/30	unclear	Rabeprazole (20mgBD), Clarithromycin (500 mg BD), Tinidazole (500 mg BD) For 1 week	Lactobacillus GG.	83.33%/80.00%
Ismail	2023	Malaysia	45/45	8 weeks	Amoxicillin (1 g BD) Clarithromycin (500mgBD) ‐ Esomeprazole (40 mg BD) Patients allergic to penicillin, metronidazole (400 mg BD) was used instead of amoxicillin. For 2 weeks	Lactobacillus reuteri DSM 17648.	91.11%/68.89%
McNicholl	2018	Spain	103/106	6 ± 2 weeks	PPI at standard doses Clarithromycin (500 mg BD) Amoxicillin (1 g BD) For 10‐days	Lactobacillus plantarum CETC7879 Pediococcus acidilactici CETC7880.	96.12%/94.34%
Moreno‐ Marquez	2021	Spain	40/40	2,4 and 6 weeks	Bismuth subcitrate potassium (140 mg 6H), Metronidazole (125 mg 6H), Tetracycline hydrochloride (125 mg 6H), Omeprazole (40 mg BD) For 10 days	Lactobacillus reuteri strains DSM 17938 and ATCC PTA 6475.	85.00%/85.00%
Naghibzadeh	2022	Iran	52/52	2 and 6 weeks	PPI, Bismuth subcitrate, Clarithromycin, Amoxicillin For 2 weeks ‐	Lactobacillus reuteri (DSMZ 17648 strain) Saccharomyces boulardii	92.31%/86.54%
Navarro rodriguez	2013	Brazil	55/52	1,2 and 8 weeks	Lansoprazole (30 mg BD) Tetracycline (500 mg BD) Furazolidone (200 mg BD) For 7 days	Lactobacillus acidophilus Lactobacillus rhamnosus Bifidobacterium bifidum Streptococcus faecium	81.82%/90.38%
Poonyam,	2019	Thailand	25/25	4week	Bismuth subsalicylate (1,048 mg BD), Metronidazole (400 mg TD), Tetracycline (500 mg 6H), Dexlansoprazole (60 mg BD) For either 7 days or 14 days.	L. reuteri DSM17938 and L. reuteri ATCC PTA6475.	96.00%/88.00%
Shavakhi	2013	Iran	90/90	2 and 6 weeks	Omeprazole (20 mg BD), Bismuth subcitrate (240 mg BD), Amoxicillin (1000 mg BD) Clarithromycin (500 mg BD) For 2 weeks	Lactobacillus casei Lactobacillus rhamnosus Lactobacillus acidophilus Lactobacillus bulgaricus Bifidobacterium breve Bifidobacterium longum Streptococcus thermophiles.	76.67%/81.11%
Srinarong	2014	Thailand	25/25	2 weeks	lansoprazole (30 mg BD), amoxicillin (1 g BD), clarithromycin (1 g OD), Bismuth (1048 mg BD) For either 7 days or 14 days.	*Bifidobacterium lactis* *Lactobacillus acidophilus* *Lactobacillus paracasei*	100.00%/96.00%
Szajewska	2009	Poland	34/32	4–6 week	Amoxicillin (25 mg/kg BD), clarithromycin (10 mg/kg BD), omeprazole (0.5 mg/kg BD) For 1 week	*Lactobacillus GG*	67.65%/68.75%
Tongtawee	2015	Thailand	98/96	4 weeks	Esomeprazole (20 mg BD), Clarithromycin (500 mg BD) or Metronidazole (400 mg TD if clarithromycin resistant), Amoxicillin (1000 mg BD) For 1 week	*Lactobacillus delbrueckii* *Streptococcus thermophilus*	90.82%/84.38%
Viazis	2022	Greece	329/335	6 weeks	Omeprazole (20 mg BD), Amoxicillin (1 g BD), Clarithromycin (500 mg TD), Metronidazole (500 mg BD) For 10 days	*Lactobacillus Acidophilus* LA‐5 *Lactiplantibacillus plantarum* *Bifidobacterium lactis* BB‐12 *Saccharomyces boulardii*	92.10%/86.87%

### 2.6 Quality Assessment

The methodological quality of trials was independently assessed by two authors using Version 2 of the Cochrane risk‐of‐bias assessment for randomized trials (Page et al. 2021). We assessed the studies to clear selection, attrition, allocation, and reporting biases. If there were any conflicts, they were resolved by mutual discussion or arbitration with an additional reviewer.

### 2.7 Statistical Analysis

The meta‐analysis followed the Cochrane Handbook for Systematic Reviews of Interventions (Cochrane Handbook for Systematic Reviews of Interventions [n.d.] Cochrane Training [Internet]). The Preferred Reporting Items for Systematic Reviews and Meta‐Analyses (PRISMA) were utilized in this meta‐analysis (Page et al. 2021). All statistical analyses were carried out using RevMan 5.3 software (Cochrane Management System) (Review Manager RevMan Computer program 2020). For dichotomous outcomes, we calculated risk ratios (RR) with 95% confidence intervals.

Statistical significance was determined using an alpha of *p* < 0.05, unless otherwise noted. When possible, meta‐analyses were conducted on intention‐to‐treat populations. Heterogeneity was assessed using the I2 statistic (Cochrane Handbook for Systematic Reviews of Interventions [n.d.] Cochrane Training [Internet]).

## 3 Results

### 3.1 Literature Search

Our full electronic database search from conception to August 2024 revealed 61 results. Following the removal of nine duplicates, 52 records remained. A later check of titles and abstracts eliminated 34 records. Remaining of the 18 ones were then procured for full‐text evaluation, and the full texts of four records were not retrieved.

**Figure 1:**
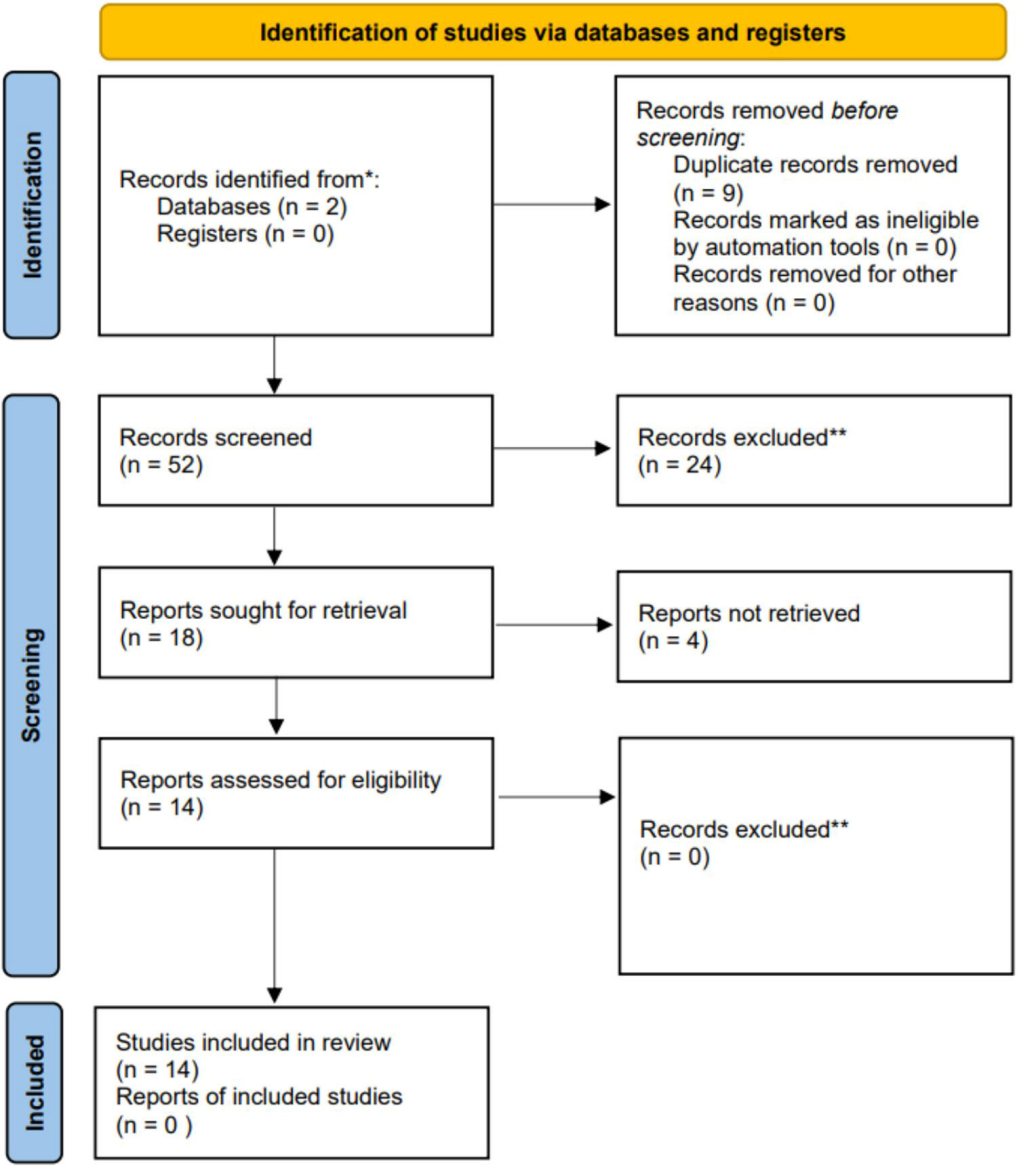
PRISMA 2020 flow diagram for the systematic review finding, reviewing, and adding papers for the systematic review and meta‐analyses are all shown in this PRISMA (preferred reporting reviews and meta‐analyses) flow diagram. The diagram consists of four main phases: identification, screening, eligibility, and inclusion. The number of studies involved in each phase are shown in figure.

We selected the remaining fourteen studies, all of which were RCTs with 2054 participants. Figure 1 comprises the inclusion process via the PRISMA flowchart.

### 3.2 Characteristics of Selected Studies

Table 1 highlights the features of the five research included, all that were published between 2001 and 2023.

### 3.3 Quality Assessment and Publication Bias

**Figure 2:**
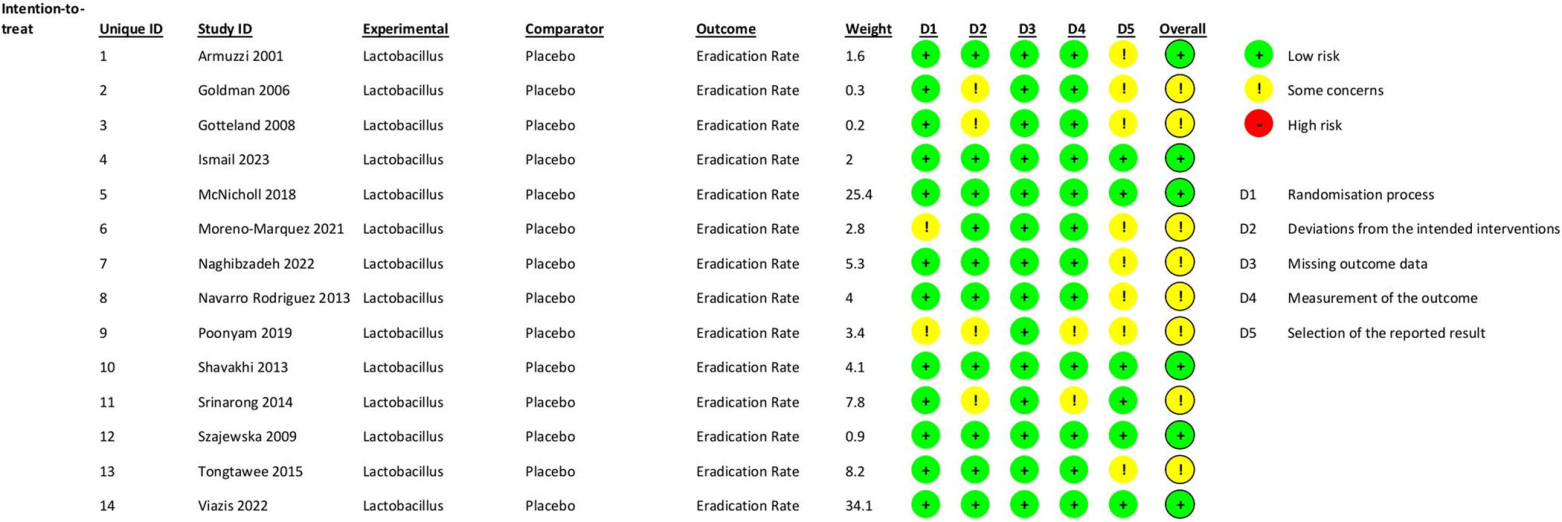
Risk of bias summary for included studies. This figure represents the risk of bias assessment for included studies. The risk of bias was evaluated by Cochrane Risk of Bias 2.0 Tool, and the results are summarized in figure. The detailed assessment for each domain is visually represented using colored circles indicating the level of risk. Green: low risk, Yellow: some concerns, Red: high risk, D1: randomization process, D2: deviations from the intended interventions. D3: absent outcome data, and D4: outcome measurement. D5: selection of the reported result.

The Cochrane Risk of Bias Tool was used to assess the potential for bias in the included studies. The majority of the 14 randomized controlled trials (RCTs) evaluating the effectiveness of LS as an additional treatment for *H. pylori* eradication had a low overall risk of bias. However, four out of 14 trials had some observed concerns, particularly in selecting reported results. The overall assessment indicates that most studies were of high methodological quality, minimizing the potential for significant bias in our analysis. A In depth result summary has been depicted in Figure 2.

**Figure 3:**
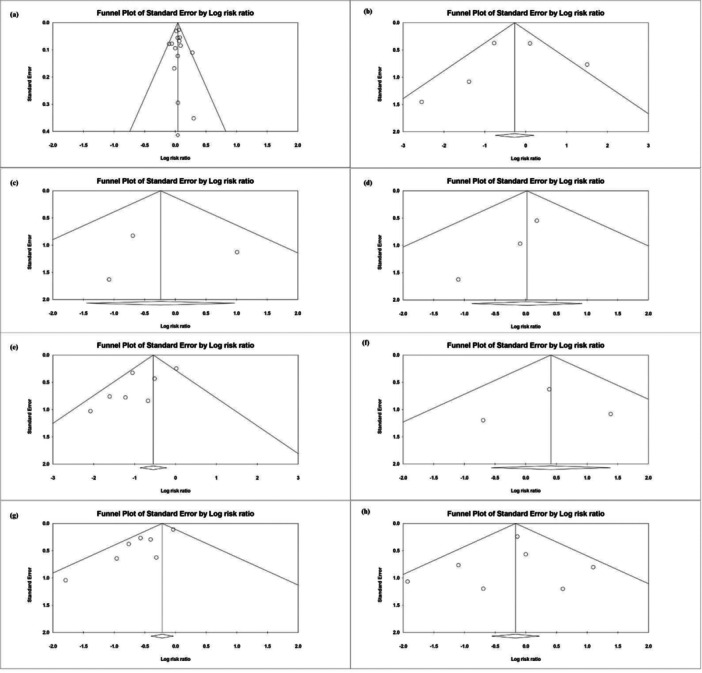
Funnel plots for Eradication rate, abdominal pain, anorexia, constipation, diarrhea, rash, taste, vomiting. (a) Funnel plot for the eradication rate. (b) Funnel plot for abdominal pain. (c): Funnel plot for Anorexia. (d) Funnel plot for Constipation. (e) Funnel plot for diarrhea. (f): Funnel plot for rash. (g): Funnel plot for taste. (h): Funnel plot for Vomiting. *X*‐axis represents the standardized mean difference (SMD) or risk ratio (RR) between the placebo and lactobacillus groups. Vertical line represents the null hypothesis, the *Y*‐axis represents the standard error. One study or data point is represented by each open circle, and the expected distribution of scales is represented by the triangle area.

**Figure 4:**
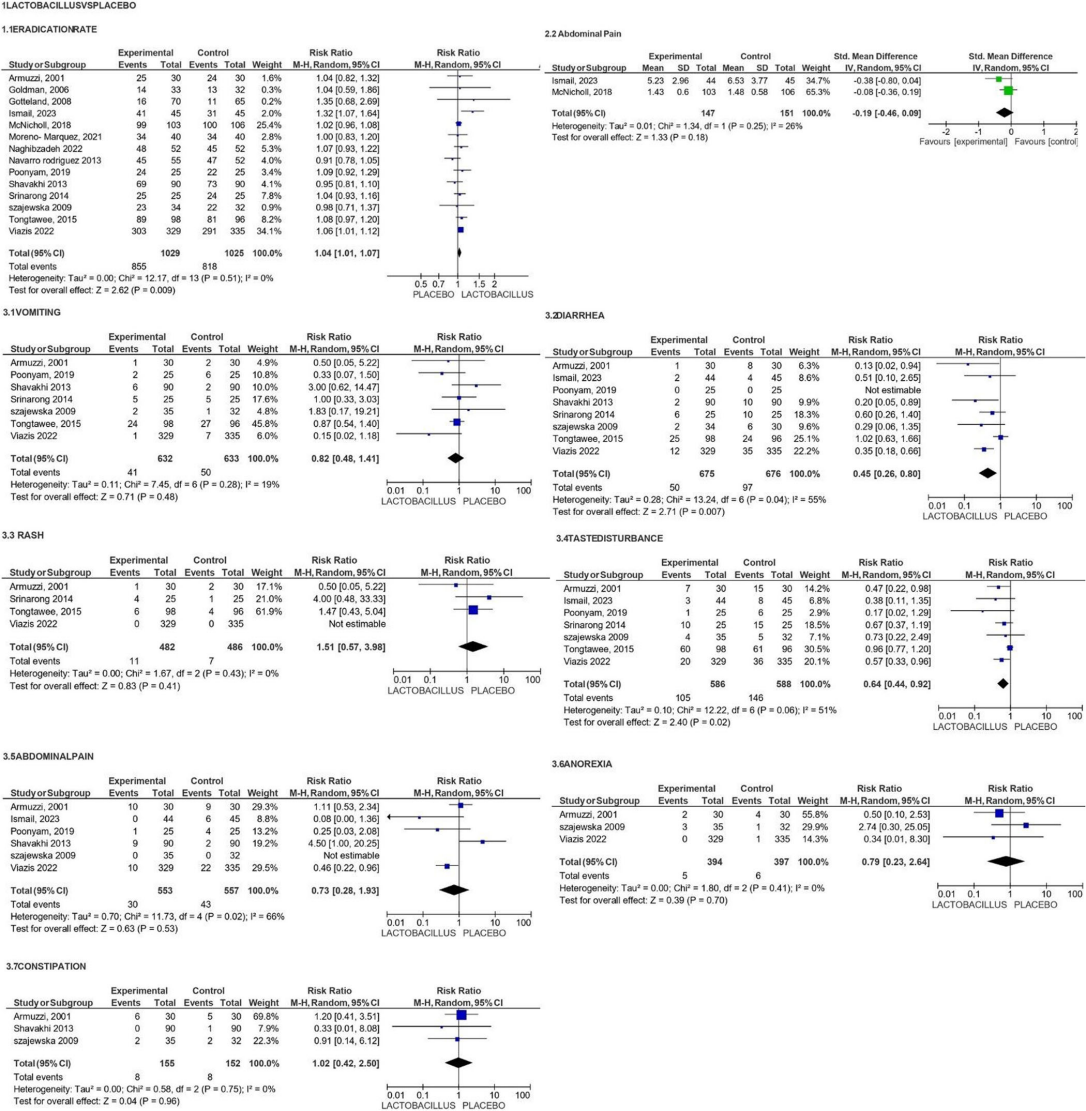
Forest plots comparing Lactobacillus Supplementation to placebo Fig. 4.0(1.1): Forest plot showing Risk Ratio for Eradication rate across 14 studies. Fig. 4.0(2.2): Forest plot showing SMD for Abdominal Pain across two studies between lactobacillus and placebo. Fig. 4.0(3.1): Forest plot showing Risk Ratio between placebo and Lactobacillus for Vomiting across seven studies. Fig. 4.0(3.2) Forest plot showing Risk Ratio for Diarrhea across eight studies. Fig. 4.0(3.3): Forest plot showing Risk Ratio for Rash across four studies. Fig. 4.0(3.4): Forest plot showing Risk Ratio for Taste Disturbance across seven studies. Fig. 4.0(3.5): Forest plot showing Risk Ratio for Abdominal Pain across six studies. Fig. 4.0(3.6): Forest plot showing Risk Ratio for Anorexia across three studies. Fig. 4.0(3.7): Forest plot showing Risk Ratio for Constipation across three studies. IV, random: inverse variance method. random effects model. Cl, confidence interval. The horizontal line represents confidence intervals for individual studies, with diamond shape representing overall effect size, *I*^2 value indicates heterogeneity.

Inverted funnel plots of the major outcomes were generated, as shown in Figure 3. The study of these plots, together with Egger's regression tests, indicated no significant evidence of publication bias (Figure 4).

### 3.4 Results of Synthesis

A total of 14 RCTs, constituting 2054 patients diagnosed with *H. pylori* (fulfilling all inclusion and exclusion criteria), have been included in the synthesis to assess effectiveness outcomes when patients received LS in addition to normal antibiotic treatment. Each RCT had data on the length of therapy collected at predetermined intervals.

In addressing the effect of LS on *H. pylori* eradication rates, the analysis demonstrated a statistically significant improvement (RR = 1.04, 95% CI [1.01, 1.07], *p* = 0.009, *I*² = 0%), (high certainty), with no heterogeneity among the studies, indicating consistency in the results.

In terms of AEs, the meta‐analysis revealed no statistically meaningful reductions in several AEs, such as vomiting (RR = 0.82, 95% CI [0.48, 1.41], *p* = 0.48, *I*² = 19%), abdominal pain (RR = 0.73, 95% CI [0.28, 1.93], *p* = 0.53, *I*² = 66%), or anorexia (RR = 0.79, 95% CI [0.23, 2.64], *p* = 0.70, I² = 0%). However, the reduction in diarrhea (RR = 0.45, 95% CI [0.26, 0.80], *p* = 0.007, *I*² = 55%) and taste disturbance (RR = 0.64, 95%CI [0.44, 0.92], *p* = 0.02, *I*² = 51%) showed some level of statistical significance.

### 3.5 Certainty of Evidence

The GRADEpro Guideline Development Tool (software) was used to assess evidence certainty (GRADEpro 2024). The information gathered on *H. pylori* clearance rates, as well as adverse effects such as vomiting, diarrhea, constipation, rash, and anorexia, was classified as high certainty due to modest confidence intervals, direct application, and precision.

Conversely, the evidence regarding taste disturbance and Abdominal pain was described as having moderate certainty. Table 2 contains a full assessment of the evidentiary certainty for each conclusion.

**Table 2:** Summary of findings comparing Lactobacillus supplementation versus placebo for Helicobacter pylori eradication therapy.

**Summary of findings for lactobacillus versus placebo for helicobacter pylori**							
Outcome and follow‐up	Patients (studies), *N*	Relative effect (95% CI)	**Absolute effects (95% CI)**			Certainty	What happens
			Placebo	Lactobacillus	Difference		
Eradication rate	2054 (14 RCTs)	RR = 1.04 (1.01 to 1.07)	798 per 1000	830 per 1000 (806 to 854)	32 more per 1000 (from 8 more to 56 more)	⊕⊕⊕⊕ High	
Vomiting	1265 (7 RCTs)	RR = 0.82 (0.48 to 1.41)	79 per 1000	65 per 1000 (38 to 111)	14 fewer per 1000 (from 41 fewer to 32 more)	⊕⊕⊕⊕ High	
Diarrhea	1351 (8 RCTs)	RR = 0.45 (0.26 to 0.80)	143 per 1000	65 per 1000 (37 to 115)	79 fewer per 1000 (from 106 fewer to 29 fewer)	⊕⊕⊕⊕ High	
Rash	968 (4 RCTs)	RR = 1.51 (0.57 to 3.98)	14 per 1000	22 per 1,000 (8 to 57)	7 more per 1000 (from 6 fewer to 43 more)	⊕⊕⊕⊕ High	
Taste disturbance	1174 (7 RCTs)	RR = 0.64 (0.44 to 0.92)	248 per 1000	159 per 1,000 (109 to 228)	89 fewer per 1000 (from 139 fewer to 20 fewer)	⊕⊕⊕○ Moderate	
Abdominal pain	1110 (6 RCTs)	RR = 0.73 (0.28 to 1.93)	77 per 1000	56 per 1,000 (22 to 149)	21 fewer per 1000 (from 56 fewer to 72 more)	⊕⊕⊕⊕ High	
Anorexia	791 (3 RCTs)	RR = 0.79 (0.23 to 2.64)	15 per 1000	12 per 1000 (3 to 40)	3 fewer per 1000 (from 12 fewer to 25 more)	⊕⊕⊕⊕ High	
Constipation	307 (3 RCTs)	RR = 1.02 (0.42 to 2.50)	53 per 1000	54 per 1000 (22 to 132)	1 more per 1000 (from 31 fewer to 79 more)	⊕⊕⊕⊕ High	
Abdominal pain	298 (2 RCTs)	—	—	—	−0.19 (−0.46 to 0.09)	⊕⊕⊕○ Moderate	

Abbreviations: CI, confidence interval; RR, risk ratio; SMD, standardized mean difference.

### 3.6 Efficacy Outcomes

#### 3.6.1 Eradication Rate

Data from 14 studies involving 2054 participants met inclusion and exclusion criteria for efficacy outcome as *H. pylori* eradication rate by LS as an adjunct to triple or quadruple antibiotic therapy. After standard antibiotic therapy combined with LS, The eradication rate improved substantially according to statistical analysis (Risk Ratio = 1.04, 95%CI [1.01, 1.07], *p* = 0.009, *I*² = 0%), indicating consistency across the included studies. These results, as displayed in Figure 1, suggest that LS provides a small but significant enhancement in *H. pylori* eradication rates compared to placebo.

#### 3.6.2 Treatment‐Related AE

##### 3.6.2.1 Vomiting

Data from seven RCTs involving 1,265 patients met inclusion and exclusion criteria for AEs related to vomiting. These were evaluated to determine the impact of LS on vomiting. The meta‐analysis found a risk ratio (RR) of 0.82 (95% CI: 0.48, 1.41; *p* = 0.48; *I*² = 19%), demonstrating that Lactobacillus reduced the risk of vomiting compared to placebo. However, the reduction was not statistically significant (*p* = 0.48). The low heterogeneity (*I*² = 19%) suggests consistency across the studies. Despite the lack of statistical significance, the evidence was rated as high certainty, reflecting confidence in the reliability of these findings.

##### 3.6.2.2 Diarrhea

Data from eight RCTs involving 1351 patients met inclusion and exclusion criteria for AEs related to diarrhea. With a RR of 0.45 (95% CI: 0.26, 0.80; *p* = 0.007), the results demonstrated a statistically significant decrease in the risk of diarrhea when using Lactobacillus supplements as opposed to a placebo. The studies showed moderate heterogeneity (*I* ^2^ = 55%). Despite this variability, the evidence was rated as high certainty, underscoring confidence in the finding that LS reduces the incidence of diarrhea.

##### 3.6.2.3 Rash

Data from four RCTs involving 968 patients met inclusion and exclusion criteria for AEs related to rash. With a RR of 1.51 (95% CI: 0.57, 3.98; *p* = 0.41), the results showed no statistically significant difference in the risk of rash between the LS group and the placebo group. The studies showed no discernible heterogeneity (*I* ^2^ = 0%), and the evidence was rated as high certainty, suggesting confidence that Lactobacillus does not significantly increase the incidence of rash.

##### 3.6.2.4 Taste Disturbance

Data from seven RCTs involving 1,174 patients met inclusion and exclusion criteria for AEs related to taste disturbance. The results indicate that LS produced a significantly reduced risk when taste disturbance was compared to placebo, with a relative risk (RR) of 0.64 (95% confidence interval [CI]: 0.44, 0.92; *p* = 0.02). The studies had moderate heterogeneity, with an *I*² value of 51%. The certainity evidence for this outcome is considered to be moderate. This suggests that while LS may reduce the incidence of taste disturbance, the variability among studies must be considered when interpreting the findings.

##### 3.6.2.5 Abdominal Pain

Six RCTs had data with 1110 patients who met inclusion and exclusion criteria for AEs related to abdominal pain. The results show that LS was associated with a relative risk (RR) of 0.73 (95% confidence interval [CI]: 0.28, 1.93; *p* = 0.53) for the incidence of abdominal pain compared to placebo. Despite the high certainty of the evidence, the confidence interval which is relatively high and *p*‐value which is insignificant, suggest that LS does not significantly affect the incidence of abdominal pain. The heterogeneity among the studies was high, with an *I*² value of 66%, indicating variability in the effect of LS on abdominal pain across the included trials.

##### 3.6.2.6 Anorexia

Data from three RCTs composed a total of 791 patients who met inclusion and exclusion criteria for AEs related to anorexia. The relative risk (RR) for anorexia in the LS group when placed against placebo was 0.79 (95% confidence interval [CI]: 0.23, 2.64; *p* = 0.70). The evidence for this outcome is considered of high certainty. The analysis revealed the difference in the anorexia incidence between the LS and placebo groups was insignificant. Additionally, there was no observed heterogeneity among the studies, with an *I*² value of 0%.

##### 3.6.2.7 Constipation

Data from three RCTs with 307 patients met inclusion and exclusion criteria for AEs related to constipation. The relative risk (RR) for constipation in the LS group observed against placebo was 1.02 (95% confidence interval [CI]: 0.42, 2.50; *p* = 0.96). The certainty of the evidence for this outcome is high. The results indicate that LS does not significantly alter the incidence of constipation compared to placebo. Observed heterogeneity seen among the studies does not exist, with an *I*² value of 0%.

#### 3.6.3 Antibiotic‐Related Gastrointestinal Symptoms

##### 3.6.3.1 Abdominal Pain

Data from two RCTs with 298 patients met inclusion and exclusion criteria for AEs related to antibiotic‐related abdominal pain. The SMD for abdominal pain was −0.19 (95% confidence interval [CI]: −0.46, 0.09; *p* = 0.18). The evidence for this outcome is moderately certain. Results suggest that LS does not significantly reduce abdominal pain associated with antibiotic use, although there is some variability in the data, as indicated by the I² value of 26%.

## 4 Discussions

Our study showed the therapeutic strength of various Lactobacillus strains as adjunct therapy used with standard antibiotic treatment for *H. pylori* eradication. Our analysis, which included 14 RCTs involving 2054 patients, showed if LS could enhance eradication rates, reduce AEs, and alleviate gastrointestinal symptoms compared to placebo.

The key findings from our meta‐analysis are as follows: LS expressed a statistically meaningful improvement in *H. pylori* eradication rates, with a relative risk (RR) of 1.04 (95% CI: 1.01, 1.07; *p* = 0.009). This suggests that while the effect size is modest, Lactobacillus may provide a beneficial adjunctive role in improving eradication outcomes. Regarding AEs, the analysis did not show meaningful reductions in most AEs, which includes vomiting, abdominal pain, and anorexia. However, LS was associated with a significant reduction in diarrhea (RR = 0.45, 95% CI: 0.26, 0.80; *p* = 0.007) and taste disturbance (RR = 0.64, 95% CI: 0.44, 0.92; *p* = 0.02). Although LS did not significantly impact the overall reduction in gastrointestinal symptoms, the effect on abdominal pain was not statistically significant, indicating variability in response.

The findings of this meta‐analysis align with a previous similar meta‐analysis from 2019 (Yu et al. 2019) which reported that LS enhances *H. pylori* eradication rates. Similar to the findings reported by Yu and colleagues, (Yu et al. 2019) improvements in eradication rates were observed. This study adds to their findings by including more recent RCTs (published after 2019), evaluating both triple and quadruple therapies. Yu and colleagues found significant reductions primarily in taste disturbance while this meta‐analysis identified additional reductions in AEs such as diarrhea. The present study also suggests that LS offers a modest yet valuable adjunct to conventional therapy by enhancing overall therapy effectiveness and mitigating certain adverse effects across diverse treatment regimens.

However, the lack of significant reduction in other AEs, such as vomiting and abdominal pain, contrasts with some studies (Wilkins and Sequoia 2017; Rau et al. 2024) that have reported broader benefits of probiotic supplementation. This discrepancy could be due to the variability in Lactobacillus strains used, dosages, and treatment durations across the included RCTs. Additionally, our analysis highlights the difficulty in assessing the relative efficacy of different Lactobacillus strains as a result of heterogeneous nature of the included studies especially in terms of lactobacillus species, dose, and duration of treatment.

Furthermore, recent taxonomic revisions have reclassified the genus *Lactobacillus* into multiple distinct genera based on phylogenetic and genomic differences (Zheng et al. 2020). This reclassification suggests that probiotic efficacy may vary among specific genera and species rather than being uniform across the former *Lactobacillus* group. However, most of the included RCTs were published before this reclassification and reported probiotics under the traditional genus name, limiting the feasibility of conducting genus‐ or species‐specific analyses. Future studies should therefore evaluate probiotic efficacy at a more detailed taxonomic level to clarify potential differences in therapeutic outcomes among distinct *Lactobacillus*‐related genera.

Our review adhered to rigorous methodological standards, including comprehensive database searches and a thorough risk of bias assessment, enhancing the findings' reliability. For *H. pylori* eradication rates and several AEs (diarrhea, taste disturbance), the evidence was rated as high certainty, reflecting confidence in the robustness of these outcomes.

All included RCTs excluded patients with chronic gastrointestinal diseases or those who had recently received antibiotic therapy, which strengthens the internal validity of our findings by minimizing potential confounders affecting *H. pylori* eradication or treatment tolerance. However, our included studies did not evaluate baseline gut microbiota composition, which could play a role in influencing both *H. pylori* pathogenesis and the therapeutic response to LS. Future studies integrating microbiome profiling may help clarify these interactions and further delineate the mechanisms underlying probiotic efficacy.

Emerging evidence suggests that the therapeutic benefits of LS may, in part, be mediated through the production of short‐chain fatty acids (SCFAs) such as acetate, propionate, and butyrate. These microbial metabolites contribute to improved mucosal integrity, modulation of gastric inflammation, and suppression of pathogenic bacterial growth, thereby potentially enhancing the overall therapeutic response against *H. pylori* infection (Ríos‐Covián et al. 2016; Tan et al. 2014). Although the included RCTs did not report SCFA measurements, understanding this metabolite‐driven interaction between probiotics and the gut environment could provide valuable mechanistic insights. Future studies integrating metabolomic profiling and SCFA quantification are warranted to elucidate the role of these compounds in mediating probiotic efficacy.

Including various Lactobacillus strains posed as a limitation in producing definitive statements regarding the therapeutic effects of specific strains. Our review is limited by moderate to considerable heterogeneity observed in certain AEs (abdominal pain and diarrhea), which may affect the robustness of the pooled estimates. Additionally, potential publication bias identified in AEs (abdominal pain and diarrhea) could further influence the findings. Future studies should aim to isolate the effects of individual strains. Variability in outcome reporting and treatment regimens across studies may influence whether the study findings apply to a broader context. Additionally, the variability of duration in the follow‐up in the studies considered for inclusion, may influence the sustainability and safety profile of LS.

## 5 Conclusion

This study shows that LS has potential to serve as a beneficial adjunct therapy for improving therapy outcomes, with a statistically significant relative risk (RR) of 1.04. While the reduction in AEs, such as diarrhea and taste disturbance, is promising, the lack of significant impacts on other AEs suggests that Lactobacillus should not replace standard treatment protocols. The objectives of further research should be to isolate the effects of specific Lactobacillus strains, standardize treatment regimens, and assess long‐term safety to clarify the role of Lactobacillus in *H. pylori* management.

## Author Contributions

**Asma Azam:** conceptualization, methodology, data curation. **Muhammad Abdul Muqtadir Qureshi:** conceptualization, methodology, data curation, formal analysis, writing – original draft. **Hafiz Shahbaz Zahoor:** conceptualization, methodology, formal analysis, writing – original draft. **Syeda Malaika Raza:** conceptualization, methodology, validation. **Muhammad Mohsin Khan:** validation. **Umaimah Naeem:** validation. **Syed Atif:** supervision, investigation, writing – review and editing. **Abdul Waheed:** supervision, investigation, writing – review and editing, project administration.

## Ethics Statement

The authors have nothing to report.

## Conflicts of Interest

The authors declare no conflicts of interest.
